# Exposure to Organic Solvents Used in Dry Cleaning Reduces Low and High Level Visual Function

**DOI:** 10.1371/journal.pone.0121422

**Published:** 2015-05-01

**Authors:** Ingrid Astrid Jiménez Barbosa, Mei Ying Boon, Sieu K. Khuu

**Affiliations:** 1 The University of New South Wales, School of Optometry and Vision Science, New South Wales, Sydney, Australia; 2 Universidad de La Salle, Health and Science Faculty, Bogotá D.C, Bogotá, Colombia; University of Waterloo, CANADA

## Abstract

**Purpose:**

To investigate whether exposure to occupational levels of organic solvents in the dry cleaning industry is associated with neurotoxic symptoms and visual deficits in the perception of basic visual features such as luminance contrast and colour, higher level processing of global motion and form (Experiment 1), and cognitive function as measured in a visual search task (Experiment 2).

**Methods:**

The Q16 neurotoxic questionnaire, a commonly used measure of neurotoxicity (by the World Health Organization), was administered to assess the neurotoxic status of a group of 33 dry cleaners exposed to occupational levels of organic solvents (OS) and 35 age-matched non dry-cleaners who had never worked in the dry cleaning industry. In Experiment 1, to assess visual function, contrast sensitivity, colour/hue discrimination (Munsell Hue 100 test), global motion and form thresholds were assessed using computerised psychophysical tests. Sensitivity to global motion or form structure was quantified by varying the pattern coherence of global dot motion (GDM) and Glass pattern (oriented dot pairs) respectively (i.e., the percentage of dots/dot pairs that contribute to the perception of global structure). In Experiment 2, a letter visual-search task was used to measure reaction times (as a function of the number of elements: 4, 8, 16, 32, 64 and 100) in both parallel and serial search conditions.

**Results:**

Dry cleaners exposed to organic solvents had significantly higher scores on the Q16 compared to non dry-cleaners indicating that dry cleaners experienced more neurotoxic symptoms on average. The contrast sensitivity function for dry cleaners was significantly lower at all spatial frequencies relative to non dry-cleaners, which is consistent with previous studies. Poorer colour discrimination performance was also noted in dry cleaners than non dry-cleaners, particularly along the blue/yellow axis. In a new finding, we report that global form and motion thresholds for dry cleaners were also significantly higher and almost double than that obtained from non dry-cleaners. However, reaction time performance on both parallel and serial visual search was not different between dry cleaners and non dry-cleaners.

**Conclusions:**

Exposure to occupational levels of organic solvents is associated with neurotoxicity which is in turn associated with both low level deficits (such as the perception of contrast and discrimination of colour) and high level visual deficits such as the perception of global form and motion, but not visual search performance. The latter finding indicates that the deficits in visual function are unlikely to be due to changes in general cognitive performance.

## Introduction

Organic solvents are a group of relatively volatile hydrocarbon compounds that are commonly used in industry (e.g., oil and petrol, paint and chemical manufacturing) and in the wider community (commercial cleaning agents and glues) to dissolve and remove materials not soluble in water [1]. Regular use of and exposure to organic solvents is known to result in neurotoxicity leading to adverse effects in the central and peripheral nervous systems (CNS and PNS respectively) including the sensory organs [2–4].

Neurotoxins may have affinity for some regions of the CNS but most have widespread effects on cellular processes involved in membrane transport in intracellular chemical reactions and the release of secretor substances [5]. Neurotoxic substances are able to cross the blood brain barrier if they have high lipid solubility. Subsequently they may interfere directly in neurological function potentially causing depression in the CNS [2,4,6–8] thereby producing adverse physiological and behavioural effects [3]. Their ability to induce adverse effects depends on their dose [9]. For example, the organic solvent perchloroethylene is known to be associated with dizziness, confusion, headache, nausea and irritation of the eyes and mucous tissues in people who are exposed to concentrations of 200 parts per million (ppm) [10]. Exposure to higher levels of PERC (more than 1500 ppm) may lead to unconsciousness and death from respiratory depression[10]. Therefore jurisdictions impose permissible exposure limits for different neurotoxins in an attempt to protect people who use these chemicals. However, the degree to which these imposed limits of exposure are effective in preventing/limiting neurotoxicity is at present unclear.

Organic solvents are commonly used in the dry cleaning industry as inexpensive cleaning agents to dissolve fat and oils from garments. Although a range of solvents are used in the dry cleaning industry, the main solvents used are the chlorinated ethenes in particular tetrachloroethene, commonly referred to as perchloroethylene and tricholoroethene (TCE). Because of the potential deleterious effects on the nervous system, the widespread use of organic solvents in the dry cleaning industry is a concern for workers and for those who live in close vicinity [11].

Exposure to organic solvents used in the dry cleaning industry has been associated with a number of visual deficits. Colour vision, particularly the blue-yellow colour vision system [12,13], and contrast sensitivity [14–17] are known to be negatively affected by exposure to organic solvents. However it is important to note that the detection of contrast and colour represent only a subset of basic visual functions and it remains unclear whether deficits in other visual functions are associated with exposure to organic solvents. The visual system is organized as a hierarchy of processing and generally divided into at least two levels: low and high whereby the lower level comprises processes that extract basic features of the image, for example object colour and contrast [18]. This low level visual processing occurs in both the prestriate areas and primary visual cortex before projecting to higher cortical areas (high level processing) which are differentially dedicated to the analysis of specialized and more complex visual behaviours such as the detection and recognition of form (e.g., shape and object discrimination), motion (global direction and speed of object motion) [19–22]. Such visual behaviours represent higher order visual judgements that are derived through the integration of basic or local image features (such as contrast and colour). While previous studies have associated exposure to organic solvents used in the dry cleaning industry with deficits in low-level visual function (colour and contrast), it remains unclear whether, and the degree to which, higher-level visual functions are negatively affected.

The neural locus or loci at which organic solvents exert their neurotoxic effect with respect to visual processing remains unclear. Animal studies have shown that injection of organic solvents results in changed retinal function as indicated by changes in the dynamics of the electroretinogram (ERG) including increased a wave amplitudes (indicator of photoreceptor health) and faster and smaller b waves (indicators of the health of the ON bipolar cells and Muller cells) [23,24]. Retinal neuronal circuitry is therefore likely to be affected which can impact on low-level visual processes. Because exposure to organic solvents produces deficits in low level functioning, this might result in flow-on disruptions in higher-level functions, which pool information from earlier cortical areas. If the organic solvent crosses into the brain, it is conceivable that cognitive processes might also be affected [18].

**Fig 1:**
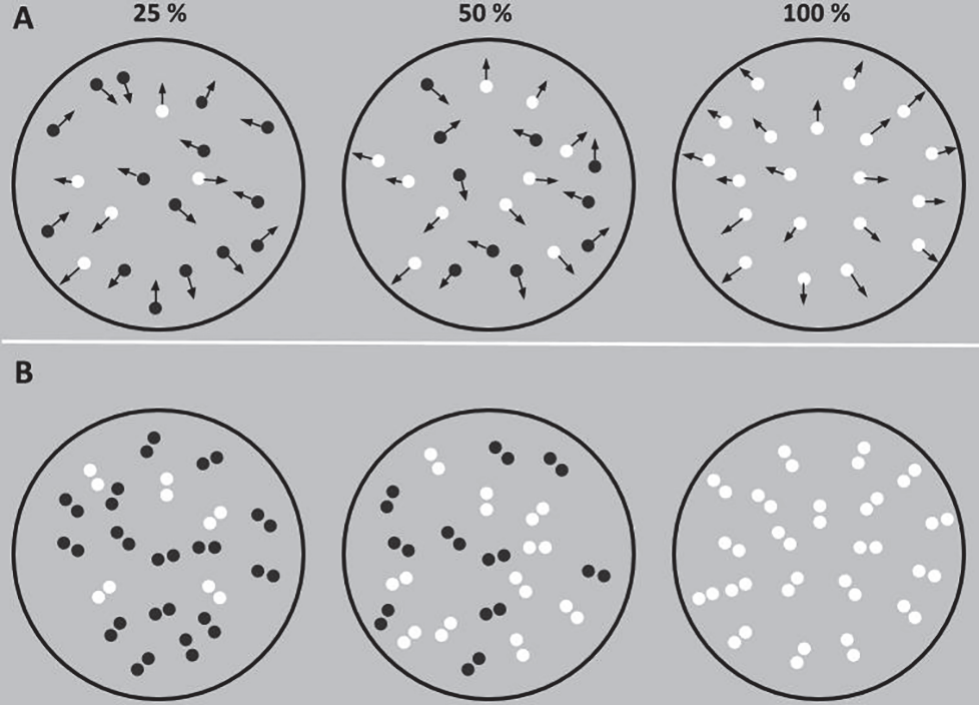
Examples of global dot motion (A) and Glass pattern (B) stimuli at different coherence levels. White dots are signal dots/dipoles which convey global motion/form information. Black dots/dipoles are noise whose motion/orientation is randomly assigned. Note that all dots were the same colour and opposite polarity dots in Fig 1 are for illustrative purposes only.

Examples of higher-level visual functions include global form and motion perception. The perception of global motion and form are ubiquitous in visual scenes, and previous studies have well demonstrated that the visual system is highly specialized for their analysis with processing occurring in cortical areas high in the visual processing hierarchy [20,22,25]. The processing of global form and motion can be assessed using dense random-dot stimuli in which either the motion of dots or their spatial placement is manipulated [26–30]. For example, global motion processing has been commonly assessed using the Global Dot Motion (GDM) stimulus [29,31,32]. The GDM stimulus is a brief movie sequence displaying a fixed number of dots moving in a common global direction (Fig 1A). To detect global motion the visual system must initially extract the local motion of dots and then integrate them to reveal the global pattern [33]. The GDM stimulus can be used to obtain a measure of sensitivity to coherent motion, by changing the ratio between dots moving in the pattern direction (signal dots), and those that move in random directions (noise dots), until the global motion can be just detected (Fig 1A). Previous studies using this paradigm have shown acute accuracy for humans performing this task, with global motion coherence detection thresholds at approximately 5–10% signal [31,34–36]. Importantly, the processing of GDM stimuli has been found to be related to the functioning of motion selective areas high in the visual pathway such as the Middle Temporal (MT) area which lies along the dorsal stream of processing [25,29].

Form processing can be assessed using Glass patterns (Fig 1B) [26–28,37,38][39] Glass patterns are commonly used to investigate form processing because their analysis by the visual system must reflect both local and global levels of computation: the orientation of local dipoles is initially extracted, and then combined at a later stage where the global form can be determined [37,38]. It is believed that the extraction of local form information reflects computation in early cortical areas such as primary visual cortex (V1) and secondary visual cortex (V2), which contains cells capable of detecting the orientation of dipoles [40,41], while integration of local form information occurs at much higher cortical areas such as V4 [42–44]. Glass patterns can be used to measure human sensitivity to global form (analogous to GDM stimuli: e.g. [29,31,34]) by changing the ratio between dipoles oriented in the pattern direction (signal dipoles), and those that have random orientations, until the global structure can be just detected (Fig 1B). Typically Glass detection thresholds are 15–25% signal [27,38,45].

The present study aimed to investigate whether exposure to occupational levels of organic solvents in the dry cleaning industry is associated with neurotoxic symptoms and visual deficits in the perception of basic visual features such as luminance contrast and colour, and for the first time, global form and motion processing. Providing clarity regarding this issue will lead to greater understanding of the broader effects of exposure to organic solvents, and the extent to which it affects visual function. In Experiment 1, neurotoxic symptoms were measured in dry cleaners exposed to occupational levels of organic solvents and non dry-cleaners, and this was compared with their performance on visual measures of contrast sensitivity, colour discrimination and global motion and Glass pattern detection.

In Experiment 2, we examined the possibility that group differences between dry cleaners and non dry-cleaners might stem from a difference in cognitive function. Exposure to organic solvents might be associated with reduction in cognitive performance however the relationship is unclear [16]. We employed a visual search task, which has been used previously to assess cognitive ability in people exposed to organic solvents and with a history of encephalopathy [16]. In this task, the reaction time required for observers to detect a letter target amongst distractors (as a function of the number of distractor elements) was quantified. It is expected that if cognitive function was impaired in dry cleaners their reaction time in visual search will be significantly slower than non dry-cleaners.

## Methods and Materials

### Study design

In the present study we report investigations of neurotoxicity and low and high- visual functions in a representative group of dry cleaners exposed to occupational levels of organic solvents. For comparison, the performance of dry cleaners was compared to an age-matched group of non dry-cleaners. The neurotoxic symptoms of both groups were assessed using the original and modified versions of the Q16 Questionnaire [46] [47], of which the original is the internationally recognized gold standard for the detection of neurotoxic symptoms [4]. For validation and to confirm that our dry cleaning sample exhibited visual system change comparable to that found previously due to exposure to organic solvents, the performance of both dry cleaners and non dry-cleaners were assessed on lower order visual tasks such as colour discrimination (Farnsworth Munsell 100 Hue test) and contrast discrimination (measuring the contrast sensitivity function), both of which have been previously demonstrated to be affected by exposure to organic solvents. Global form and motion using GDM and Glass pattern stimuli in dry cleaners and non dry-cleaners to establish whether exposure to occupational levels of organic solvents is associated with deficits in these higher order perceptual tasks. Finally, visual search was assessed.

### Participants

Participant inclusion and exclusion criteria were as follows: The inclusion criteria for dry cleaners in the study were: 18 to 40 year old adults who worked for at least one year in dry cleaning establishments which use organic solvents in their cleaning processes and had normal or corrected to normal visual acuity (better than 0.1 log MAR). Dry-cleaner participants were required to have worked at one or more of the various stations in the dry cleaning process (e.g: washing, ironing, quality control, clothes delivery). This age range was selected to exclude age-related or maturational changes in vision from complicating the analysis. The inclusion criteria for non dry-cleaners (controls) were: 18 to 40 year old adults who worked in technical level jobs and had never worked at dry cleaning establishments, never used any organic solvents in their work, had normal or corrected to normal visual acuity (better than 0.1 log MAR), never lived with anyone who works in the dry cleaning establishment and had never lived in the same building as a dry cleaning establishment, because indirect exposure can cause neurotoxicity leading to contrast sensitivity and colour vision changes [11,48]. All participants were recruited from the metropolitan region of Sydney, Australia.

The exclusion criteria for both the dry cleaners and non dry-cleaners were: congenital colour vision deficiencies (assessed with the Ishihara pseudoisochromatic plate test), systemic and neurological diseases unrelated to environmental toxins, macular diseases, and corneal or crystalline lens opacities.

The dry cleaning group (n = 33) had a mean age of 31.85 years (SD 6.86) and had been exposed to organic solvents for a median of 3.5 (range 1: 16.5) years full time employment. They worked an average of 8.5 (SD 2.5) hours per day, washed, ironed and folded a median of 100 (range 120) garments per day. The control group (n = 35) had a mean age of 29.14 years (SD 3.9). All dry cleaners were assessed at their workplaces. Written Informed consent was obtained from all participants.

The Human Research Ethics Committee University of New South Wales had approved the Informed Consent Form. After consenting, the participants were provided with a signed copy of the Informed consent. The original signed copy of the informed consent form is kept by the investigators in a secure place. The tenets of the Declaration of Helsinki were observed throughout.

## Procedure

### Assessment of Neurotoxicity

Neurotoxicity was assessed in a sample of dry cleaners (and compared to non dry-cleaners) to provide an indication of the degree to which exposure to occupation levels of organic solvents used in the dry cleaning industry affected visual system function in our sample. Neurotoxicity was assessed using the neurotoxic symptoms questionnaire Q16 [46,49]. The Q16 questionnaire is commonly used to monitor the early effects of neurotoxic exposures in the working population and has been internationally recognized as the gold standard test for the detection of neurotoxic symptoms by the World Health Organization [4]. It contains 16 questions on symptoms commonly described by workers exposed to solvents such as: “I have a short memory”; “I often have a painful tingling in some part of my body”; “I feel that I have less sensitivity or a complete loss of sensitivity in some parts of my arms or legs” graded for ‘yes or no’ agreement. The overall score, which represents the number [47] and extent [46] of positive reports of symptoms, provides an indication of the degree of neurotoxicity. Although morphologic and classical toxicological methods may be used to provide evidence of neurotoxicity [50,51], the Q16 has gained acceptance and is regarded as an excellent method of symptom reporting for neurotoxicity as well as a valid screening tool for neurotoxicity [4]. This questionnaire has been widely validated and used in different neurotoxic studies [52–57], and has been recommended for screening for neurotoxicity [58–60].

### Clinical Ocular and Visual Examination

Eye health and vision screenings were conducted by a registered optometrist. A Log Mar visual acuity chart was used to assess distance visual acuity and a Jaeger reading card (Latham & Philips Ophthalmic, Ohio USA) was used to evaluate near visual acuity (40cms). Slit lamp biomicroscopy (Care optical SLM-J/1/2/2E/2L, China) was used to evaluate the presence or absence of lens opacities using the lens opacities classification system III (LOCS III)[61]. Direct ophthalmoscopy was used to assess ocular health. The participants were refracted using auto refractor equipment (Topcon KR-3000, Japan) and those who needed refractive correction were optically corrected with untinted lenses. Colour vision was evaluated for red- green congenital colour vision deficiency, using the Ishihara test (24 plates). Participants who did not meet the inclusion criteria were excluded from participating in the study.

### Behavioural Assessments

Stimuli were generated on an Apple Macintosh Mac Book Pro computer. All psychophysical tests were programmed within MATLAB (The Math-works, Inc version 2010) using the Psychophysics Toolbox [62,63]. The laptop screen (resolution 1920x1080) was used to present stimuli which was gamma corrected prior to data collection.

All psychophysical tests were conducted monocularly; using the eye with the worst best-corrected visual acuity (VA). Note that although both eyes had normal vision, one eye may have slightly better vision than the other eye. Worst VA was used because both the eyes of participants were corrected in terms of refractive error and had no pathology based on clinical observation to be included in the study, so that any deficit in VA would be likely to have a neural basis upon which the neurotoxic action of OS, if any, may exert their influence.

Participants viewed the stimuli from a distance of 70 cm in a dimly lit-room and performed each test once. Practice trials were given prior to data collection to ensure task familiarity and reduce procedural learning effects. All participants completed all tests.

To examine the contrast detection ability of dry cleaners and non dry-cleaners, the contrast sensitivity function (CSF) was measured. This function provides a description of contrast sensitivity, ability to detect luminance differences, over a wide range of spatial frequencies. The CSF was measured using an oriented Gabor patch. The Gabor patch was presented in the middle of a grey screen (luminance of 55 cd/m^2^) and was oriented either to the left or to the right by 45 degrees from the vertical. The task of the observers was to indicate the orientation of the Gabor pattern. If the Gabor pattern’s orientation could not be perceived, the participant was asked to guess. Therefore, a two alternative orientation forced choice paradigm was used together with a 3 down 1 up psychophysical staircase procedure. In this procedure, the Michelson contrast of the stimulus, coinciding with the amplitude of the Gabor stimulus, is increased if the observer makes one error of judgement and is decreased if the observer makes three consecutive correct judgements of orientation in a row. This procedure was designed to converge to the 79% correct performance level. Initially the starting contrast of the stimulus was 0.8 and the step size was 0.08. After the first and subsequent reversals the step size was halved. After the third reversal the step size was 0.01 and remained at this value until the end of the staircase trial. The bit-stealing algorithm outlined by Tyler (1997) was used to generate a look-up table to achieve a luminance resolution of up to 12 bits (i.e., 4096 gray-scale levels). The staircase lasted for 6 reversals and the average of the last 4 reversals was averaged to estimate the contrast detection threshold. No feedback was given to indicate the correctness of response. The staircase procedure was at spatial frequencies of 0.5, 1.0, 2.0, 4.0, 8.0 and 16.0 cycles per degree (cpd) in randomised order.

As discussed earlier, global dot motion perception was assessed using GDM stimuli which consisted of a 10-frame movie displaying 500 moving (6°/s) white-dots (70 cd/m^2^ radius 0.06) on a grey background (55 cd/m^2^). On the first frame dots were randomly placed within the stimulus area with a radius of 5° (thus the total dot density was approximately 13 dots/deg^2^). On each and subsequent frames a proportion of these dots moved coherently (signal dots) while the remaining dots moved in random directions (noise dots). Signal dots moved in directions to convey radial motion, which has been shown to be selectively processed in higher cortical areas [25,64,65]. Signal and noise dots were randomly selected without replacement from the entire pool of dots every frame to prevent observers from tracking individual dots. Each movie was shown for 50ms with no inter stimulus interval. Thus the total duration of the stimulus was 500ms.

Global form perception was assessed using Glass patterns which were generated using the same procedures as those used to create GDM stimuli. However, there was only one stimulus frame. The Glass pattern stimulus consisted of 500 dots but dots were locally grouped to produce 250 dot-pairs (or dipoles) with a dot pair separation of 0.25°. Dipoles (Fig 1) were locally oriented to convey radial form structure (signal dipoles) or were randomly oriented (noise dipoles). The Glass pattern stimulus was presented for 500ms.

Both GDM stimuli and Glass patterns were presented to the observer in a two-alternative forced choice design. For GDM stimuli one interval contained global radial motion, while the other interval consisted only of randomly moving dots. The task of the observer was to determine the interval containing the signal pattern. For Glass patterns, signal and noise only patterns were presented in two intervals, and the task of the observer was to judge the interval containing global radial form. For both GDM and Glass pattern stimuli, the order of signal and noise intervals were randomized from trial to trial and separated by an interval of 250ms in which the screen was blank. A psychophysical staircase procedure that converged on the 79% correct performance level was used to modify the signal level of both stimulus types from trial to trial. On the first presentation the proportion signal of the stimulus was 0.6 and the step size was 0.08. The step size was halved after each reversal; after the 3rd reversal the step size was 0.01 and remained at this level until the end of the staircase run. The staircase lasted for 8 reversals and the average of the last 4 reversals provided an indication of the coherence threshold.

The Farnsworth Munsell Hue 100 (FM100) was used to assess hue discrimination ability of participants. The test consists of 85 caps of different hues which observers must arrange in order according to hue similarity. Hue discrimination may be abnormal in the case of congenital colour vision deficiencies with participants making characteristic confusions of certain pairs of colours. Hue discrimination may also be abnormal in the case of acquired colour vision deficiencies, due to abnormalities of the colour pathway arising from toxicity or pathology. Acquired losses are usually not as sharply defined as congenital colour losses therefore the FM100 test is well suited to assessing non predictable colour errors [66]. In accordance with standard colour testing [67], this test was conducted in a closed room and illuminated using a daylight fluorescent lamp (Phillips cool daylight tube, capable of a luminous intensity range of 0.8 to 90 cd/m^2^) with a colour temperature of 6,500K and colour rendering index of 98[68]

## Results

### Neurotoxicity

**Fig 2:**
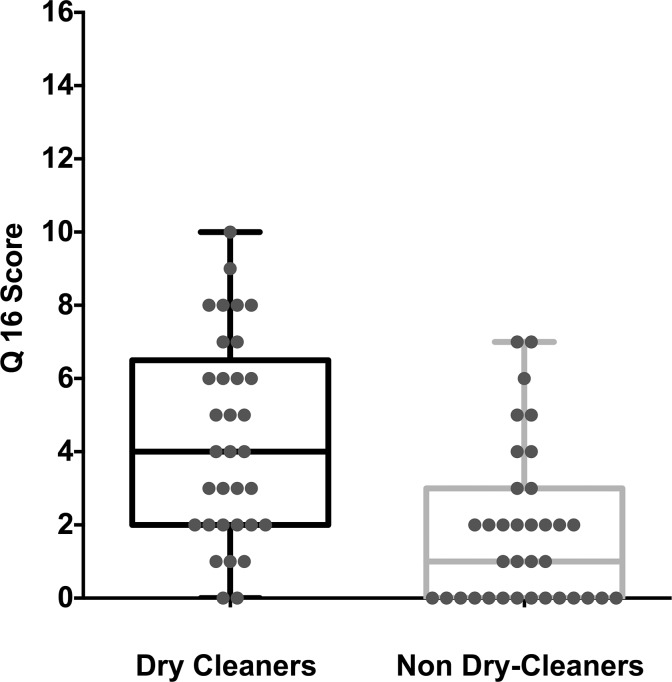
Box and whisker plot of the Q16 score for dry- cleaners and non dry-cleaners.

The original Q16 scores are presented and shown as a box and whiskers plot in Fig 2. Both the original and modified Q16 scores showed similar trends however for greater ease of comparison with previous studies, only the original Q16 scores are presented in the results. The average Q16 score for dry cleaners was 4.33 (±2.76SD) while non dry-cleaners reported a score of 1.82 (±2.12SD). Note that Q16 scores and their variability are consistent with previous studies that have adopted this questionnaire to examine neurotoxicity in groups exposed and not exposed to organic solvents. For example in a review of 19 papers by Ihrig, Triebig and Dietz [57] the mean Q16 scores and mean standard deviations for groups exposed to organic solvents was 4.07 and 2.85 respectively, while for those not exposed (to organic solvents) the mean Q16 score and mean standard deviation were 2.4 and 2.6 respectively.

Because the Q16 response is binary (yes—no responses) and nominal in scale the non parametric Mann Whitney-U test (two-tailed) was used to compare the mean Q16 score between dry cleaners and non dry-cleaners in the present study. Drycleaners were found to report significantly higher (Mann-Whitney U:265, p<0.0001) numbers of neurotoxic symptoms on average than non dry-cleaners (Fig 2) This result indicated that the dry cleaners who participated in the present study had higher than normal levels of neurotoxicity on average, consistent with previous studies [46,54,69].

According to the WHO, the Q16 score can also be used as a means to screen individuals who might have neurotoxicity. Based on the data provided by Hosgstedt et a. (1984) [70] an individual with a Q16 score of 6 or more are likely to be adversely affected by exposure to organic solvents. For our dry cleaning group 12 out of 33 participants have Q16 scores of 6 or greater. Surprisingly 3 out of 35 non dry-cleaners also met this criterion, which suggests that some non dry-cleaners displayed a high number of symptoms that are not attributable to exposure to organic solvents. The finding of some high Q16 scores in a group not exposed to organic solvents is consistent with Ihrig et al.(2001) [55] who noted the variability associated with the Q16. It is important to emphasize that the Q16 provides a general measure of symptoms commonly associated with neurotoxicity, but the test is not exhaustive nor are the measured symptoms exclusive to the condition.

### Contrast sensitivity

**Fig 3:**
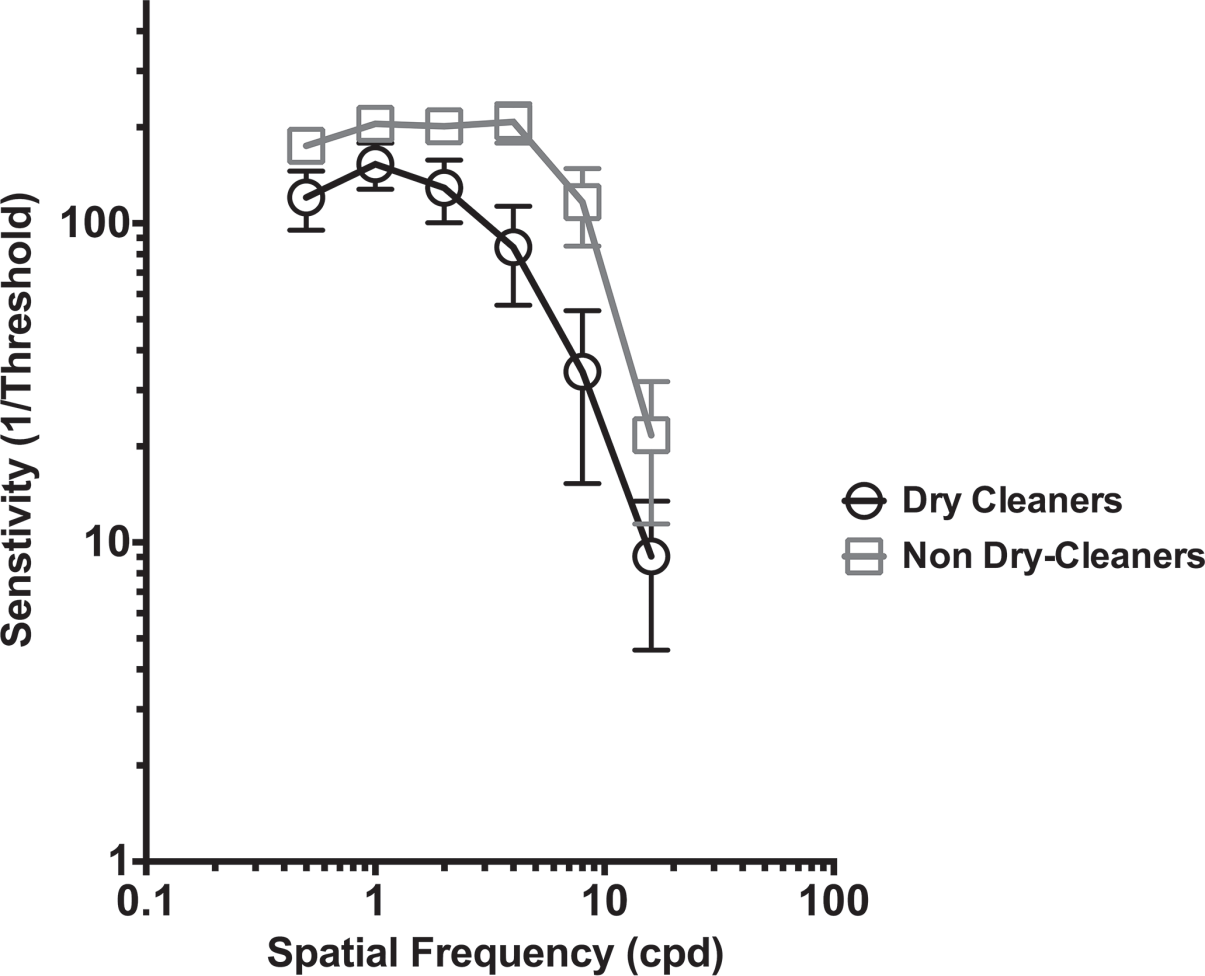
Log contrast sensitivity plotted as a function of spatial frequency for dry cleaners and non dry-cleaners. Error bars represent 95% confidence intervals.

The average CSF for dry cleaners (circles) and non dry-cleaners (squares) are plotted in Fig 3. Error bars represent 95% confidence intervals. A repeated measures two-way ANOVA was performed on these data observed a main effect of both spatial frequency (F (5, 330) = 86.71, p<0.0001) and group (F (1,66) = 39.33, p<0.0001). However a significant interaction effect (F (3, 330) = 7.40, p<0.0001) was also reported which indicated that there was a significant difference in the shape of the CSF between groups. In Fig 2, contrast detection resembled the classic CSF function with sensitivity greatest between 3–8 cpd and gradually decreasing with increasing spatial frequency [71]. However, the CSF function for dry cleaners was lower than for non dry-cleaners. Holm-Sidak Post-hoc tests (corrected for multiple comparisons) were performed to determine group differences at different spatial frequencies. Significant between-group differences (p<0.05) were observed at all spatial frequencies assessed. This result indicated that dry cleaners had significantly poorer contrast sensitivity across a broad range of spatial frequencies relative to non dry-cleaners.

### Colour Discrimination

**Fig 4:**
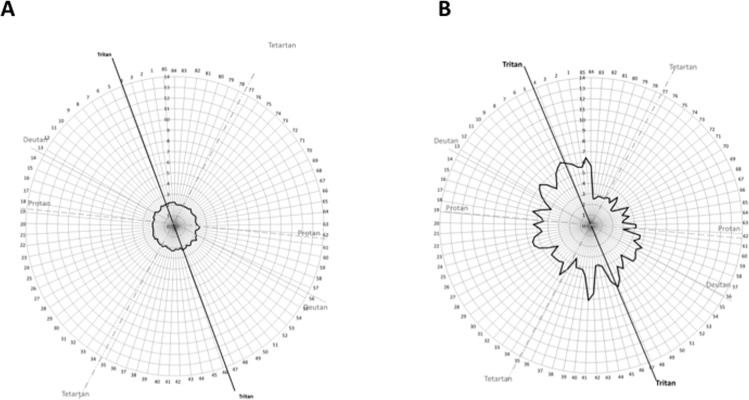
The average of Hue discrimination results of participants is plotted as a black, solid line. Polar graph of non dry-cleaners (4A); polar graph of dry cleaners (4B).

Average hue discrimination results for dry cleaners and non dry-cleaners are shown in a polar plot of error score as a function of cap number in Fig 4A and 4B. On the plot, scores of 2 indicates no error in terms of ordering of immediately adjacent colour caps in the series of 85 caps. Higher scores on the plot indicate colours of greater difference in hue were confused as being similar by the observers. It can be seen that the non dry-cleaners made very few errors on average whereas the dry cleaners had larger numbers of errors and greater colour confusion. Lines indicating typical colours which are confused by people with congenital colour vision deficiencies due to problems with the L-, M- and S- photoreceptors are indicated by the lines labelled “Protan”, “Deutan” and “Tritan” respectively. To provide a normalised overall measure of performance, the square root of the total error score (√TES) was derived for each observer in both groups [72,73]. We find that the √TES values for non dry-cleaners were normal (7.03 (SD±0.21) and are according to the age normal values (Table 1) established by Kinnear and Sahraie (2002) [73]. However, the √TES of dry cleaners was significantly higher (√TES 12 (SD±2.72) (p<0.01) compared to non dry-cleaners. This indicated that dry cleaners had greater difficulties with hue discrimination than non dry-cleaners.

**Table 1:** The New Farnsworth Munsell Hue 100 test norms of normal observers using √TES (Kinnear and Sahraie, 2002).

Age	Mean √TES
5	19.1
6	17.5
7	15.5
8	14.0
9	12.4
10	11.2
11	10.2
12	9.5
13	9.0
14	8.6
15	8.1
16	7.5
17	7.0
18	6.7
19	6.6
20	6.7
21	6.7
22	6.8
30–39	7.3
40–49	8.1
50–59	9.5
60–69	10.7
70–79	12.3

A Fisher exact test (with Yates’s continuity correction) was conducted to determine whether the proportion of participants who made tritan-like errors (typically associated with exposure to organic solvents [13,74]) differed between the two groups of dry cleaners and non dry-cleaners. The Fisher exact test indicated that dry cleaners made more tritan type errors than non dry-cleaners (X2 (2, N = 33) = 11.71, p<0.01). We note that no individual in the non dry-cleaner group made tritan-like errors whereas 16 out of 33 dry cleaners made tritan like errors. As mentioned in Methods, lens clarity was assessed and it was found that there was no significant difference between the LOCS III grading of the crystalline lenses in dry cleaners and non dry-cleaners (Mann-Whitney’s U test, p = 0.09). Additionally, the median and range of scores of transparency of the crystalline lens indicate clarity for both groups. This is an important assessment as tritan-like errors have been associated with senescence of the crystalline lens [75] which would indicate an optical rather than a neural explanation for the colour vision deficit. The results indicate that crystalline lens changes are unlikely to account for changes in color vision therefore a neural, rather than optical impairment may be inferred.

### Global Form and Motion

**Fig 5:**
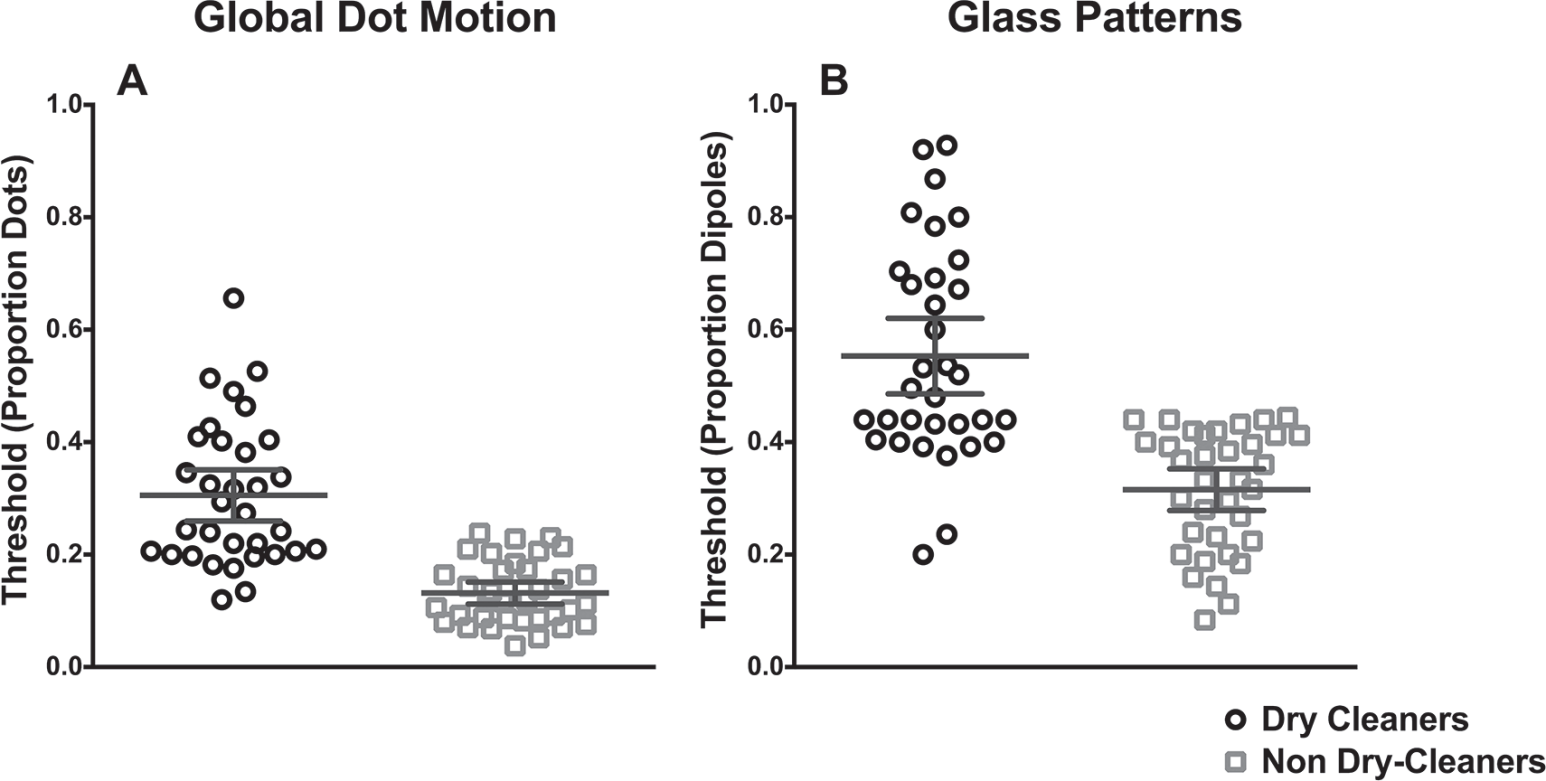
Coherence thresholds (proportion signal dots/dipoles) for the detection of GDM (A) and Glass patterns (B). In each plot individual thresholds are clustered for dry cleaners (squares) and non dry-cleaners (circles). Indicated in both figures are horizontal lines representing the group mean. Error bars represent 95% confidence intervals.

Thresholds for global motion and form detection are shown in Fig 5A and 5B respectively. In each plot the proportion of signal dots/dipoles required to detect radial motion is plotted separately for dry cleaners (squares) and non dry-cleaners (circles). Data points represent individual thresholds and the mean for each group is given as horizontal lines within each data cluster. Error bars represent 95% confidence intervals. In Fig 5A, the average GDM thresholds for dry cleaners and non dry-cleaners were 0.35 and 0.132 respectively. A t-test (two-tailed) indicated that dry cleaners on average had significantly higher GDM thresholds (t (66) = 7.3, p<0.0001) than non dry-cleaners.

A similar trend is also observed with global form detection. As shown in Fig 5B, the average threshold for detecting, Glass pattern structure for dry cleaners were 0.55, while for non dry-cleaners the average threshold was lower at 0.3. A t-test confirmed that dry cleaners had significantly higher thresholds than non dry-cleaners (t (66) = 6.31 p <0.0001).

It is possible that high level visual deficits in global form and motion processing may be due to flow on effects from a reduction in the perception of contrast. Higher thresholds for global form and motion might be observed in dry cleaners because the stimulus is perceptually lower in contrast (the contrast attenuation model, [76]) and consequently the global pattern might be harder to detect. To investigate this possibility we determined whether a relationship exists between the contrast sensitivity of observers and their global motion and form detection. To provide a single and overall measure of contrast sensitivity the “area under the contrast sensitivity curve” was determined for dry cleaners and non dry-cleaners and this value was compared with their GDM and Glass pattern thresholds in a linear regression analysis. This analysis revealed that contrast sensitivity was not significantly correlated with global motion (slope = 0.015, F (1, 33) = 0.002, p = 0.997) or form detection (slope = 3.337, F (1, 33) = 0.480, p = 0.493) for non dry-cleaners. There was also no significant relationship between contrast sensitivity and the ability to detect global motion (slope = -1.58, (F (1, 31) = 0.502 p = 0.48) and form (slope = -1.41, (F (1, 31) = 0.216, p = 0.645) for the dry cleaners.

As both Glass patterns and GDM stimuli comprised of small dots it is possible that a relationship might exist between contrast sensitivity to high spatial frequencies and detection of these global form and motion patterns. However, there was no correlation between GDM and Glass pattern thresholds and sensitivity to the highest spatial frequency (16cpd) for both non dry-cleaners (GDM: slope 0.094, F(1,33) = 0.786, p = 0.381; Glass pattern: slope -0.014, F(1,33) = 1.687, p = 0.202) and dry cleaners (GDM: slope 0.002, F(1,31) = 0.117, p = 0.0.734; Glass pattern: slope 0.003, F(1,31) = 0.163, p = 0.688).

These results demonstrated that elevated global form and motion thresholds in our subjects were not related to the measured reduction in contrast sensitivity (even at high spatial frequencies), but rather direct deficits in both motion and form processing. This is consistent with previous studies that have noted that the effect of contrast on global form and motion perception is primarily observed when stimulus contrast is well below 0.1 [77,78]. Even taking into account the contrast attenuation model [76], 0.1 stimulus contrast is much lower than the levels of contrast perception which were measured in this study.

## Experiment 2: Visual search performance in dry cleaners and non dry-cleaners

Experiment 1 showed that dry cleaners exposed to occupational levels of organic solvents displayed more neurotoxic systems and performed worse in visual tasks that assessed low and higher level visual function. However, it should be noted that the effect of organic solvent exposure is certainly not exclusive to visual processing [58,59,79,80] but is observed in a number of neural functions. It has been observed that exposure to organic solvents might lead to cognitive impairments, such as deficits in attention, short-term memory, associative learning and psychomotor speed [80,81]. Therefore it is possible the group differences between dry cleaners and non dry-cleaners reported in Experiment 1 might be attributed to a general cognitive deficit rather than one that is a specific to visual processing. In particular, exposure to organic solvents might lead to impairment in cognitive function and affects the way in which the observer *responds* to, rather than *sees* the visual stimulus. In Experiment 2, we addressed this issue by examining the performance of dry cleaners and non dry cleaners who participated in Experiment 1 on a visual search task.

Visual search is a perceptual task requiring attention that typically involves an active scan of the visual environment for a target stimulus (e.g., a letter) placed amongst distractor stimuli (e.g., dissimilar letters). According to Broadbent (1958) [82], visual search can be used to measure cognitive function and represents how visual attention is deployed to various parts of the visual field. The time taken to perform this task is commonly used as a means of quantifying this cognitive process as well as a means of differentiating the type of search [18,83–88] In the case of the target and distractors being dissimilar in form (e.g. P vs O), parallel search processes are employed whereas target and distractors with similar forms (e.g. E vs F) employ serial search strategies [86,89]. Importantly, a letter identification visual search task has been used by Näsänen, Kaukianinen et al. (2005)[16] to identify changes in cognitive function in patients diagnosed with encephalopathy and a history of chronic exposure to organic solvents.

## Methods

**Fig 6:**
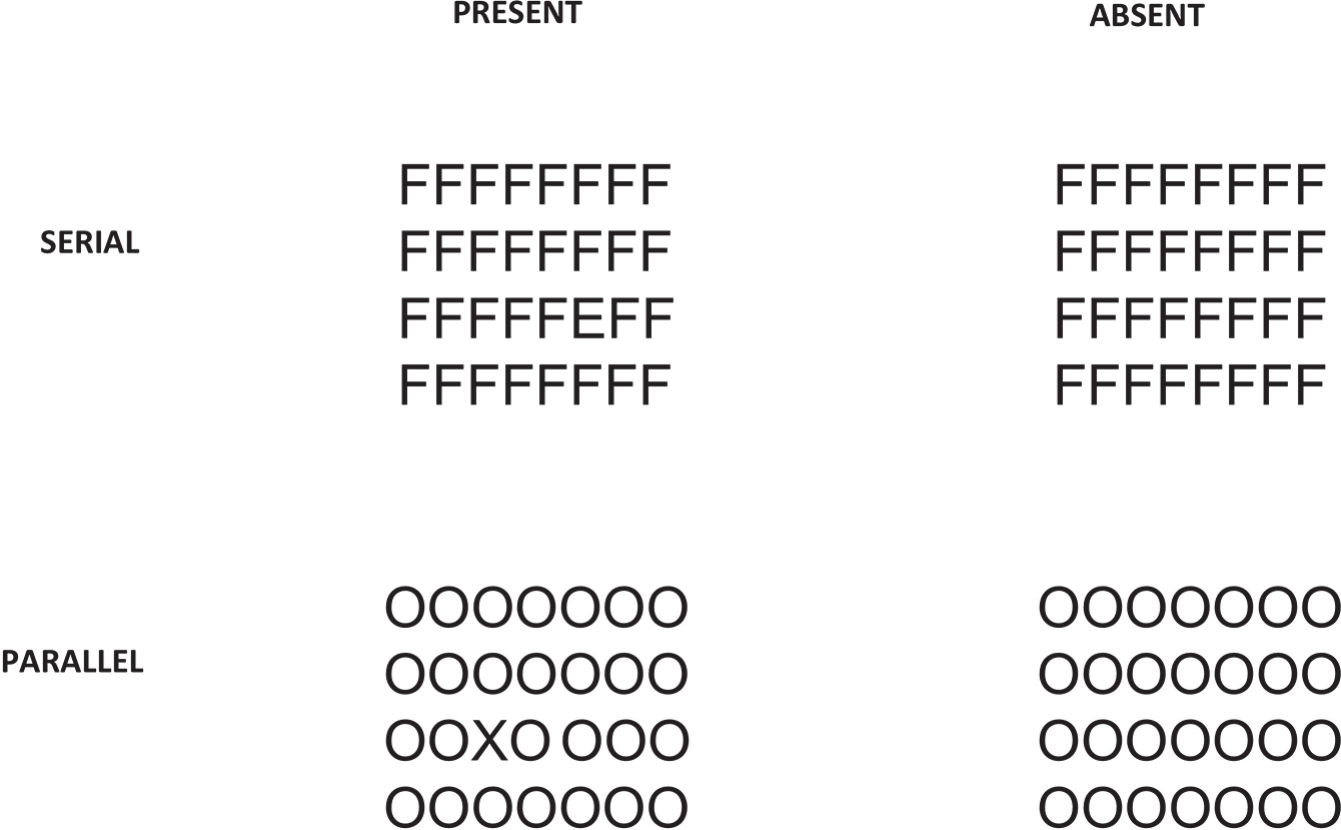
Examples of the letter visual search stimulus used in Experiment 2.

The stimuli used in the visual search task in the present study were black letters (Arial font, size 16) presented on a white background (luminance 30 cd/m^2^). The task of the observer was to search and identify the letter “X” (in parallel search type) among the “Os” (Fig 6A) and the letter “E” (in serial search type) among “Fs” (Fig 6B). The maximum duration of the stimulus presentation was 8 seconds; if the observer did not respond during this period that trial was represented at a later point in the testing sequence. For both conditions the task of the observer was to search and indicate (by pressing keys on the keyboard) whether the target was absent or present in the stimulus.

Visual search performance was expressed in reaction time for correct responses only (on average 88.6% for target present conditions, 88.5% for target absent conditions in serial search, and 100% for both absent and present conditions in parallel search) and corresponded to the minimum time taken for the observer presses the key on the keyboard once the target was detected. The response signalled the onset of the next presentation after a 500 ms delay. An auditory signal was given as feedback for incorrect responses.

Reaction times were measured for parallel and serial search and absent and present conditions 20 times, with all conditions interleaved. Thus there were 80 trials in total. This was repeated for stimulus set sizes of 4, 16, 36, 64 and 100 elements respectively.

## Results

**Fig 7:**
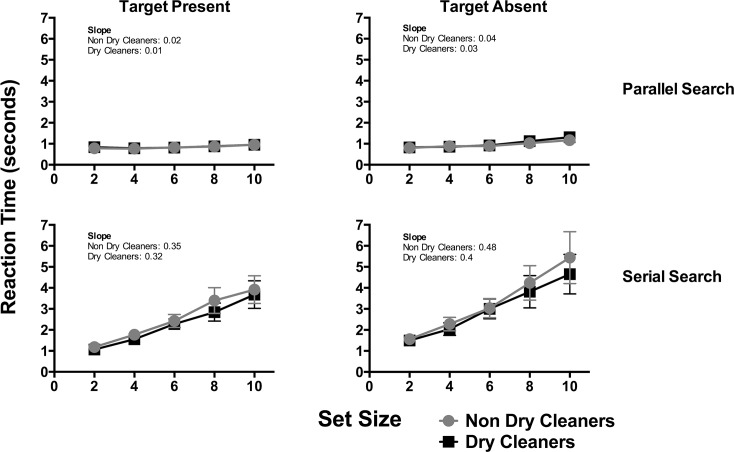
Mean visual search reaction times (in seconds) plotted as a function of the set size. Data for dry cleaners (squares) and non dry-cleaners (circles) are plotted for conditions in which the target was present or absent and for serial and parallel search. Error bars represent 95% confidence intervals. In each plot the slope of the line of best fit is also shown for the two groups.

Mean reaction times (in seconds) required to identify the presence and absence of the target for serial and parallel search conditions are shown in Fig 7. In these plots reaction time data for dry cleaners (gray-circle) and non dry-cleaners (black-squares) are shown as a function of the number of distractor elements. Error bars signify 95% confidence interval. Linear regression analysis was performed on these data to provide an estimate of the slope of the best-fit line (using GraphPad Prism 6), which provided a measure of visual search efficiency in terms of the seconds per item.

For parallel search, a two-way ANOVA comparing reaction times between groups (dry- cleaners and non dry-cleaners) and different set sizes were performed separately for target present and absent conditions. This analysis did not show a main effect of set size for target present (F(4,365) = 1.39, p = 0.236), which indicated that reaction times did not change with set-size, which indicated that the distractors did not impair the detection of the target stimulus, presumably of a ‘pop-out’ effect. Note that the slope of the line of best fit for both groups was approximately 0. A significant effect of set size was observed for target absent conditions (F(4,365) = 10.86, p<0.0001), however, the increase in search time per item is negligible at 0.003 and 0.005 seconds per item for dry cleaners and non dry-cleaners respectively. An effect of set size might be attributed to the fact that the target was absent from the stimulus (i.e., there was no ‘pop out’ effect), and at larger set sizes, participants might spend more time to confirm the absence of the stimulus. Importantly though, there was no significant group difference between dry cleaners and non dry-cleaners for target present (F(1,365) = 1.07, p = 0.302) and absent (F(1,365) = 1.70, p = 0.193) conditions. This analysis showed that the reaction times for both groups in parallel search were the same, which suggests that exposure to organic solvents was not associated with the ability to perform parallel visual search.

For serial search, a main effect of set size was observed for both target present (F(4,356) = 59.30, p<0.0001)) and absent (F(4,356) = 37.89, p<0.0001)) conditions. Indeed, increasing the number of distractor elements affected the detection of the target stimulus with search reaction times significantly increased by approximately 0.28 (F(1, 173) = 107.6, p<0.0001) and 0.26 (F(1, 198) = 124.1, p<0.0001) seconds per item for non dry-cleaners and dry cleaners respectively in target present conditions. In target absent conditions, serial search increased by 0.39 (F(1, 173) = 77.24, p<0.0001) and 0.32 (F(1, 198) = 73.62 p<0.0001) seconds per item for non dry-cleaners and dry cleaners respectively. However, similar to with parallel search, there was no main effect of group for both target present (F(1,365) = 2.71, p = 0.105) and absent (F(1,365) = 2.25, p = 0.135) conditions. These outcomes suggest that reaction times required to detect the presence or absence of a target were the same for dry cleaners and non dry-cleaners. Importantly, these findings indicate that the dry cleaners that participated in the present study were not impaired, relative to non dry-cleaners, in their ability to perform serial visual search.

In summary Experiment 2 showed that both dry cleaners and non dry-cleaners performed similar in both serial and parallel search conditions. This suggests that exposure to organic solvents did not lead to an impairment in the cognitive function as measured using visual search. Accordingly, while organic solvent exposure might affect the ability of the observer to detect the visual properties of the stimulus (i.e., its contrast, colour, form and motion) it did not impair how they *responded* to the stimulus. In this context, the results of Experiment 1 represent specific impairment in visual function and not general cognitive change.

## General Discussion

In Experiment 1 we reported that exposure to organic solvents used in the dry cleaning industry are associated with impairments in both low level and high levels of visual processing and that dry cleaners suffer from higher levels of neurotoxicity compared to non dry-cleaners of similar age as revealed by the Q16. Significantly, the present study demonstrated that impairments in the perception and detection of global motion and form, higher level visual functions, are also associated with exposure to organic solvents.

In Experiment 2 we noted that the difference in visual performance is unlikely due to a difference or change in cognitive function due to exposure to organic solvents. We note that visual search performance in dry cleaners and non dry-cleaners were similar in a letter visual identification task. These results are not consistent with those of Näsänen, Kaukianinen et al. (2005)[16], which may be explained by a difference in the characteristics of the participants of each study. As mentioned, Näsänen, Kaukiainen et al. (2005) [16] examined patients diagnosed with encephalopathy and a history of chronic exposure to organic solvents on a letter identification visual search task. They found that patients performed poorly in both parallel and serial visual search tasks, hence it was suggested that exposure to organic solvents were associated with impairments in the speed of visual information processing, and/or limitations in attentional capacity. Because their participants experienced two conditions, it is possible that the neuropathy related to the encephalopathy, might explain their poorer visual search performance rather than their exposure to organic solvents. Another potential explanation may be differences in the age of the participants in each study. Näsänen and colleagues examined individuals chronically exposed to organic solvents who were aged between 51 and 62 years of age, a cohort which was older than the dry-cleaner group examined in the present study (mean age: 31.85 ±6.86) which might suggest that either age or long term exposure might also be factors, which are discussed next.

It might be expected that the changes in low and high level visual function are correlated with the number of years of exposure, and the level of neurotoxicity as measured by the Q16. However, previous studies suggest that there is little relationship. For example, Lacerda, Gomes et al (2011)[66] [90] reported no correlation between the visual performance on contrast sensitivity and colour perception with years of exposure to organic solvents. Also, Costa, Salgueiro et al (2012)[17], who examined a group of gasoline workers exposed to organic solvents, did not find a significant correlation between number of working years exposed to organic solvents and contrast sensitivity. Collectively, these results suggest that years of exposure to organic solvents may not be a contributing factor in changes in visual performance. To provide an indication of the effect of years of exposure in our dry cleaning group we examined the correlation between the number of years of exposure (for the dry-cleaner group), with performance on the four visual tasks measured in the present study. Similar to the findings of Lacerda Gomes et al (2011)[66] and Costa, Salgueiro et al (2012)[17], no significant correlations were found between the number of years of exposure to organic solvents and contrast sensitivity (AUC, r = 0.077, p = 0.667), colour discrimination (√TES, r = -0.076, p = 0.675), GDM (r = -0.322, p = 0.067) and Glass pattern (r = 0.217, p = 0.225) thresholds. This finding suggests that number of years of exposure may not be a contributing factor to visual task performance, rather individuals develop visual deficits after initial exposure to organic solvents [91]. According to Cullen and Redlich 1995 [92] and Iyaniwura 2004[93], host susceptible factors such as metabolic variation, nutritional status, inmunogenetic factors and neuroepithelial function determine the specific way that a person reacts to a toxin; as a result each individual possesses their own factors that determine the level of protection from intoxication by a chemical substance. It may be speculated that people who remain in the industry may share certain qualities such as innate level of physiological protection from organic solvents, and that people who are more susceptible to the toxic effects of organic solvents may voluntarily choose to leave the industry early therefore any dose-response relationship, if any, may be obscured.

Ihrig, Nasterlack et al (2003) [56] also noted that there was no correlation between the Q16 score and colour perception in painters exposed to organic solvents. We conducted a similar analysis and consistent with the findings of Ihrig, Nasterlack et al [56], we report no correlation between the Q16 score of observers and visual performance on contrast sensitivity (AUC, r = 0.033, p = 0.855), colour discrimination (√TES, r = -0.193, p = 0.288), GDM (r = -0.262, p = 0.146) and Glass pattern (r = 0.240, p = 0.184) thresholds. A possible explanation for this is that the Q16 was not designed to specifically assess visual function, but rather general symptoms of neurotoxicity. Consequently, it could be argued that the Q16 might not be specifically sensitive in indicating visual deficits from exposure to organic solvents. A worthwhile future step would be the development of a vision specific questionnaire that seeks to directly assess visual neurotoxic symptoms. This could then be validated against behavioural tests similar to those used in the present study, and provide a more accurate measure of *visual* neurotoxicity.

The present study replicated previously reports of lower order visual deficits in contrast detection and hue discrimination associated with exposure to organic solvents, particularly those used in the dry cleaning industry [15,17,94–97]. The finding of poorer contrast sensitivity in dry cleaners is consistent with the findings of Altman and Bottger (1990) who reported that workers exposed to occupational levels of organic solvents in the microelectronics industry have lower contrast sensitivity, especially at low spatial frequencies of 0.8; 1.0 and 2.0 cpd, compared to non dry-cleaners [95]. Our results also agree with Mergler et al (1991) who noted that the CSF was affected by chronic solvent exposure at 3, 6 and 12 cpd spatial frequencies, which fall within the same range of the measurement of the present study[98]. Other studies that measured contrast sensitivity in workers exposed to organic solvents or solvent mixtures, reported a reduction at all spatial frequencies >1.5 cpd [14,17,98–104]. The results in this study are not in agreement with Schaper, et al. (2004) who did not observe any contrast sensitivity loss in a group of workers exposed to the organic solvent toluene [105]. However, differences in methodology could explain the discrepancy between the findings of the present study and those of Schaper et al. and Lacerda et al [66,105]. For example, Schaper et al., (2004) used the Pelli-Robson Contrast Sensitivity Chart, which might lack the precision of our computer-based test in which the contrast step size was small and controlled using appropriate psychophysical procedures. Additionally, the Pelli-Robson contrast sensitivity chart does not assess contrast sensitivity at specific spatial frequencies, but rather provides an overall measure of contrast sensitivity, which might mask losses at particular spatial frequencies.

Several studies have demonstrated that workers exposed to organic solvents have colour vision problems [11,13,17,66,101,106–111]. However, the mechanism for colour vision changes is unclear. Organic solvents appear to more often result in tritan deficits [112], rather than protan or deutan deficits, as was found in the present study. It has been thought that the relative scarcity of blue cones, or short wavelength sensitive cone photoreceptors (approximately 10% of all photoreceptors) combined with an increased sensitivity to chemical induced damage [113] may lead to more tritan (blue-yellow) deficits in vision. The results of the present study indicated that crystalline lens changes are unlikely to account for the noted tritan colour impairment. Both groups had clear crystalline lenses which suggests that impairments in hue discrimination in dry cleaners is likely to be due to neurological rather than optical deficits such as poor transmission of short wavelengths of light.

Previous studies have reported that the visual system detects form with a high degree of efficiency, requiring the stimulus to contain only 0.15–0.2 proportion of signal dipoles in order to detect glass structure [38,45,114] and 0.5–0.1 moving signal dots for GDM detection. The results of non dry-cleaners in the present study were consistent with previous studies with normal observers. However, we find that dry cleaners had poorer global motion and form detection performance, with thresholds on average double those of non dry-cleaners. This finding shows for the first time that exposure to organic solvents is associated with reduction in the detection and perception of global form and motion. The significance of the present study is that exposure to organic solvents not only leads to deficits in low level function (such as the detection of contrast as reported here and elsewhere) which are related to retinal and neural function up to V1, but also extends to higher order behavioural judgments of global motion and form [9,115,116]. Whether this impairment reflects a flow on effect from lower visual areas affected by organic solvents or direct change in higher cortical areas remains unresolved as the psychophysical approach of the present study is unable to differentiate between these two possibilities. Future research could help resolve this uncertainty; for example electrophysiological studies of retinal, primary visual cortex and higher cortical areas would be informative in further understanding the site(s) of neurotoxicity in the visual system as electrophysiological processing could be directly visualized at each site using electroretinogram or visual evoked potential studies with active electrodes overlying the primary and higher visual areas respectively.

It is important to note that these observed effects were found in dry cleaners despite the regulation of their exposure to organic solvents, indicating that current occupational levels of exposure are impacting on the health of the nervous and visual systems of dry cleaners. This should prompt re-evaluation of circumstances of exposure to organic solvents and work practices by the dry cleaning industry, employers and the wider community.
